# A Within-Subject Multimodal NIRS-EEG Classifier for Infant Data

**DOI:** 10.3390/s24134161

**Published:** 2024-06-26

**Authors:** Jessica Gemignani, Judit Gervain

**Affiliations:** 1 Department of Developmental and Social Psychology, University of Padua, Via Venezia, 8, 35131 Padua, Italy; judit.gervain@unipd.it; 2 Padova Neuroscience Center, 35131 Padua, Italy; 3 Integrative Neuroscience and Cognition Center, Université Paris Cité & CNRS, 75006 Paris, France

**Keywords:** NIRS-EEG co-registration, MVPA, classification, newborns

## Abstract

Functional Near Infrared Spectroscopy (fNIRS) and Electroencephalography (EEG) are commonly employed neuroimaging methods in developmental neuroscience. Since they offer complementary strengths and their simultaneous recording is relatively easy, combining them is highly desirable. However, to date, very few infant studies have been conducted with NIRS-EEG, partly because analyzing and interpreting multimodal data is challenging. In this work, we propose a framework to carry out a multivariate pattern analysis that uses an NIRS-EEG feature matrix, obtained by selecting EEG trials presented within larger NIRS blocks, and combining the corresponding features. Importantly, this classifier is intended to be sensitive enough to apply to individual-level, and not group-level data. We tested the classifier on NIRS-EEG data acquired from five newborn infants who were listening to human speech and monkey vocalizations. We evaluated how accurately the model classified stimuli when applied to EEG data alone, NIRS data alone, or combined NIRS-EEG data. For three out of five infants, the classifier achieved high and statistically significant accuracy when using features from the NIRS data alone, but even higher accuracy when using combined EEG and NIRS data, particularly from both hemoglobin components. For the other two infants, accuracies were lower overall, but for one of them the highest accuracy was still achieved when using combined EEG and NIRS data with both hemoglobin components. We discuss how classification based on joint NIRS-EEG data could be modified to fit the needs of different experimental paradigms and needs.

## 1. Introduction

Functional Near Infrared Spectroscopy (fNIRS) and Electroencephalohraphy (EEG) are two of the most popular neuroimaging methods in the developmental neurosciences. fNIRS is a functional neuroimaging technique that, by means of red and near-infrared light, measures relative concentrations of oxygenated and deoxygenated hemoglobin in the illuminated tissues, thanks to the fact that at the wavelengths of red and near-infrared light the two hemoglobin components display different absorption spectra [1]. EEG is a non-invasive technique of electrophysiological imaging that measures the electric fields produces by neuronal activity in the brain [2].

The two methods offer very different, yet complementary strengths [3]. EEG directly measures the neural activity of the brain and offers excellent temporal resolution as it captures relevant changes in electrical brain activity unfolding in the millisecond range. By contrast, it has poor spatial resolution as electric potentials generated at the neural sources mix and add up at the scalp measurement points. By contrast, fNIRS relies on the hemodynamic correlates of neural activity, and thus provides precise spatial localization, since the signal measured at each channel location is estimated to be arising exclusively from the head volume underlying the channel’s source and detector [4]. However, NIRS offers a lower temporal resolution because the hemodynamic response develops slowly, peaks within several seconds, often 5–10 s or even more in young infants, after the onset of stimulation, and requires an additional 10–20 s to return to baseline [5].

Both techniques are commonly employed with newborns and infants as they are perfectly safe and non-invasive. Because they yield complementary information, their simultaneous registration is highly desirable because it provides high temporal resolution as well as spatial localization. Further, the two signals do not interfere with one another, and EEG electrodes and NIRS optodes can easily be inserted into the same headgear.

Despite this highly promising possibility, very few NIRS-EEG co-registration studies have been conducted with infants [6,7,8,9,10]. This is partly due to methodological challenges [11] and the fact that the relationship between the electrophysiological response and the accompanying hemodynamic response is not fully understood, even in adults. Before NIRS-EEG co-recording can become a routine methodology in developmental neuroscience and clinical practice, work is first needed to develop methodological and analysis practices to analyze and interpret the EEG and the NIRS signals together.

In the current work, we aim at introducing a multivariate and multimodal classifier in an attempt to leverage features from both signals. In particular, the workflow we present is illustrated using data from five subjects, newborn infants, presented with auditory stimuli of different nature: human speech and monkey vocalizations. Both NIRS and EEG have long been employed in studies testing linguistic abilities as soon as at birth, in newborns [12,13,14,15,16,17]. The goal of this work is to show how a multivariate NIRS-EEG classifier can be developed to discriminate multivariate (i.e., multichannel) and multimodal patterns of brain activity elicited by two auditory conditions carrying linguistic and non-linguistic content.

Multivariate pattern analysis (MVPA) techniques have gained increasing recognition in recent years. For example, they have been shown to be more sensitive than the corresponding univariate analysis in discriminating NIRS responses elicited in infants by stimuli that differed in content [18]. Similarly, MVPA also successfully discriminated NIRS responses to spoken and sign language in infants [19]. Using EEG, Bayet and colleagues [20] successfully employed MVPA on a selection of electrodes to discriminate the orientation of visual stimuli in children.

From the technical point of view, applying MVPA techniques to EEG is less complicated than to NIRS, thanks to the larger number of epochs typically available in an EEG study. Due to the slower time scale of the hemodynamic response measured by NIRS, the number of available blocks is typically more limited, making it difficult to set up a classifier that has enough data for both training and testing [21]. The different temporal dynamics of the two techniques also poses a challenge for setting up an MVPA framework for concurrent NIRS-EEG. In particular, given the different number of trials available in the two modalities within the same experiment, it is challenging to build a feature matrix that employs both types of data, and to leverage their correlation.

However, classification based on combined NIRS and EEG data is desirable as they provide a more complete picture of neural activity and possibly better classification accuracy. Indeed, classification based on EEG data alone often yields suboptimal accuracy because of low signal-to-noise ratio (SNR), poor spatial resolution, and the challenge of selecting the most appropriate features among many potentially relevant ones [22]. Combining it with another modality, like fNIRS, is thus highly desirable as it can enhance decoding power. In order to develop an NIRS-EEG classifier, several methods have been proposed for merging data from the two modalities [23]. Specifically, combining them can take place at the level of the results, i.e., between the outcomes of two separate classifiers, one for each modality, or at the feature level, with a single classification routine that works on a multimodal feature matrix.

Illustrating the approach of combining the results of two separate classifiers, Fazli and colleagues [24], for instance, implemented a two-step classification in which fNIRS measurements were used in a first step to predict the performance of an EEG-based brain computer interface (BCI), implemented in a second step. This approach yielded high classification accuracy and has since been replicated in other studies [25]. The method combining NIRS and EEG features into a single classifier has been implemented in several different ways, such as concatenating them [26], transforming them [27], or optimizing feature selection in some way [22,28].

Critically, none of these methods have been tested on infant NIRS-EEG data, making our study the first one to tackle this challenge. Classification of infant data is inherently more challenging than adult data, even when using a single modality, given that infant data often have lower overall signal quality, a lower number of available trials, and higher inter-individual variability due to maturational differences, generally resulting in more modest classification accuracies [29].

In this work, we merge the feature matrices of the two modalities and run a single classification routine over them. The data come from a study that used an innovative experimental design [9], in which the presentation of EEG trials is nested within that of larger NIRS blocks, thus resulting in a direct temporal correlation between the two signals. We extract and concatenate features from the two modalities while keeping their temporal inter-dependency. We use the resulting NIRS-EEG feature matrices to classify auditory stimuli using MVPA at the individual subject level, employing a modified version of a toolbox recently published to carry out EEG-based MVPA [29]. We illustrate the method on data from five different newborn infants.

## 2. Materials and Methods

### 2.1. Participants

Five healthy full-term newborns (3 females, 2 males; gestational age between 39 w and 41 w; APGAR scores: 9/9/10 or above; normal otoacoustic emissions test; mean weight 3230 g) were tested on their day of birth at the Maternity Ward of the Casa di Cura di Abano Polispecialistica e Termale. Parents gave informed consent prior to participation. The study was approved by the Ethics Committee of the Province of Padua (DG 1251-2022). The data were collected as part of a larger, ongoing study.

### 2.2. Stimuli and Experimental Design

To create the stimuli, 27 Italian sentences were selected from the Italian Antelmi subcorpus [30] of the CHILDES database [31]. Selected sentences included declaratives and interrogatives that ranged in length between 1 and 4 syllables (e.g., *ho capito* “I understood”; *perchè no?* “why not?”; *si, dai* “yes, come on”; *che cosa?* “what?”). All sentences were recorded by three different Italian female speakers in a child-directed manner. Sentences ranged in duration between 0.4 and 1 s. Mean pitch was 255.2 Hz. Twenty-seven baboon “wahoo” vocalizations [32] were also selected from the database available in [33]. The baboon vocalizations were chosen so as to match the Italian sentences in duration at the item level. All sound files were equated in intensity to 70 dB using PRAAT. The sentences and vocalizations showed no significant difference in duration (t = −0.607, *p* = 0.55; Table 1), or in mean pitch (t = 1.356, *p* = 0.18).

**Figure 1:**
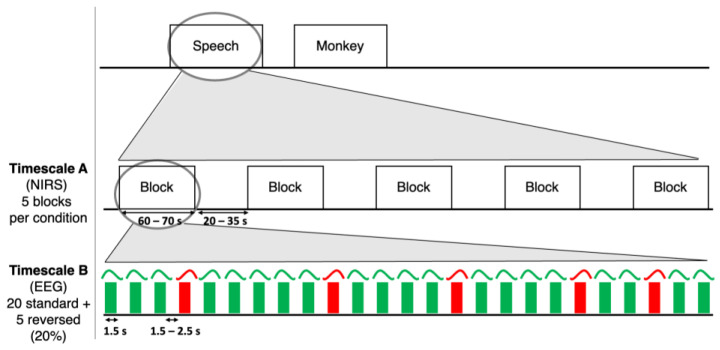
The experimental design of the study. Timescale A describes the timing of presentation of NIRS blocks, while Timescale B describes the timing of presentation of EEG trials, with green ones representing the presentation of standard sentences/vocalizations and red ones representing the presentation of deviant, or time-reversed, sentences/vocalizations.

Both in the Speech and the Monkey Vocalization conditions, two types of stimuli were created: standard, forward going stimuli described above, and deviant, time-reversed stimuli. These latter were generated in PRAAT by time-reversing each standard, forward item. Time-reversed sounds were then used as deviant trials for an odd ball (or mismatch) type of EEG presentation (Figure 1, Timescale B). However, the mismatch response, i.e., the comparison between standard and deviant trials, will not be of relevance in the present work, as the NIRS-EEG MVPA was carried out on the standard trials only.

The experiment consisted of 5 blocks per condition (Figure 1). Blocks had a duration of between 60 s and 70 s and were spaced at intervals randomly jittered between 20 s and 35 s. Their order of presentation was randomized with the constraint that blocks of the same condition could not be presented consecutively more than twice. Each block contained 25 repetitions of the same sentence in the Speech condition, or vocalization in the Monkey Vocalization condition. Of the 25 repetitions, 20 sentences/vocalization were standards, the remaining 5 were deviants, i.e., time-reversed. Deviants occurred randomly among the 25 repetitions, and never consecutively. The whole duration of the experiment was 17 min. This experimental design, which nests shorter trials arranged in an odd ball or mismatch design, typical of EEG paradigms, within longer blocks suitable for NIRS, was recently introduced by Cabrera and Gervain [9].

### 2.3. Procedure

Infants were tested during sleep while lying in their crib in a quiet room of the Maternity Ward of the Padua University Hospital. Their mothers were present in the room throughout the whole testing session.

**Figure 2:**
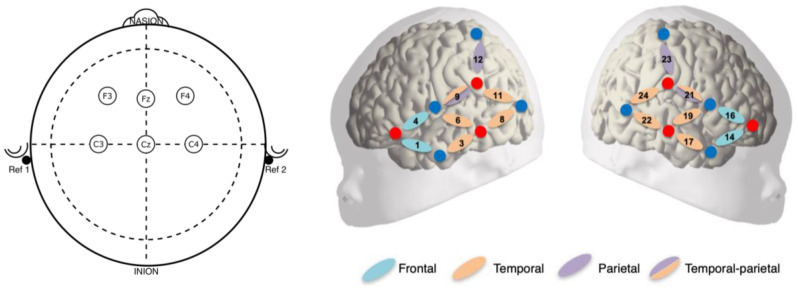
Configuration of the NIRS-EEG cap. (**Left**) EEG positions; (**Right**) NIRS positions. Red circles indicate light sources, blue circles indicate detectors. The anatomical localization of the resulting 16 channels, indicated in the legend, is described in [34].

NIRS data were recorded using an NIRx NIRSport 2 system (NIRx Medizintechnik GmbH, Berlin, Germany). This machine uses pulsated LED lights at 760 nm and 850 nm to record the NIRS signal at a sampling rate of 13.568 Hz, with a source-detector separation of 3 cm. The optical probes, 6 sources and 8 detectors, were inserted into a stretchy cap (EasyCap) in a configuration yielding 16 channels (8 per hemisphere), probing temporo-parietal areas (Figure 2, right). EEG recording was performed with a Brain Products actichamp EEG amplifier (Brain Products GmbH, Munich, Germany) and active electrodes. Six active electrodes were embedded in the same cap at 10–20 sites F3, Fz, F4, C3 and C4, and Cz (Figure 2), referenced to the two mastoids. The signal was recorded at 500 Hz.

Stimuli were presented through two loudspeakers elevated to the height of the newborn’s crib and positioned at a distance of 1 m from the newborn’s head at an angle of 30°. A computer running E-Prime delivered the stimuli and sent time stamps to the NIRS and EEG machines.

### 2.4. Data Analysis

#### 2.4.1. fNIRS Pre-Processing

NIRS light intensity measures were first converted to optical densities. Then, a motion artifact correction routine was carried out, using the temporal derivative distribution repair (TDDR) algorithm introduced by [35]. Corrected optical densities were then converted to oxygenated (HbO) and deoxygenated (HbR) concentration changes, using the modified Beer–Lambert Law, using the following absorption coefficients (μ_a_, mM^−1^ × mm^−1^): μ_a_ (HbO, 760 nm) = 0.1496, μ_a_ (HbO, 850 nm) = 0.2526, μ_a_ (HbR, 760 nm) = 0.3865 and μ_a_ (HbR, 850 nm) = 0.1798. The product of the optical pathlength and the differential pathlength factor was set to 1, so that the resulting concentration changes are expressed in mM x mm.

**Figure 3:**
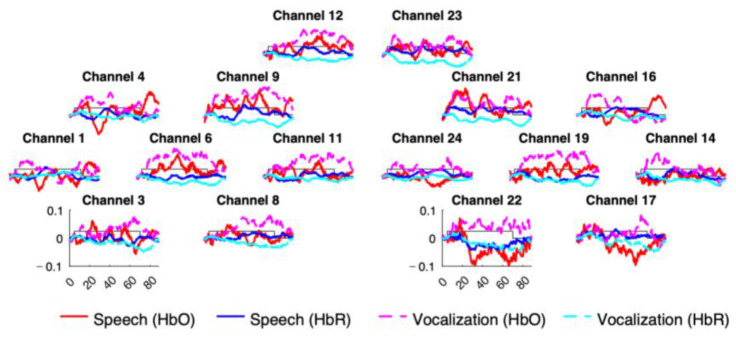
Grand-averaged hemodynamic responses elicited by speech sentences and monkey vocalizations.

Concentration changes were then band-pass filtered using a digital fft (fast fourier transform) filter, between 0.001 and 0.7 Hz. Finally, a routine was carried out to check the data quality in each channel-block pair. In particular, a block in a given channel was rejected if the light intensity reached the saturation value (1.2 V), if the block contained motion artifact, or both. Motion artifacts were defined as signal changes larger than 0.1 mM × mm over 200 ms. Finally, for the non-rejected blocks, a baseline was linearly fit between the means of the 5 s preceding the onset of the stimulation and the 5 s starting 20 s after the offset of stimulation. The 20 s window was chosen to allow enough time for the HRF to return to baseline. Figure 3 shows the obtained grand average hemodynamic responses.

#### 2.4.2. EEG Pre-Processing

**Figure 4:**
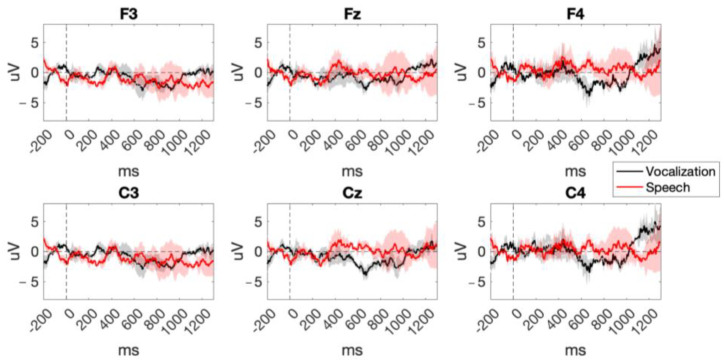
Grand-averaged ERPs averaged over the standard trials of speech and monkey vocalizations. Shaded error bars represent the standard error of the mean.

EEG data were pre-processed using functions of the EEGLAB toolbox (version 2022.1 [36]) as well as custom scripts. Continuous EEG data were first band-pass filtered between 1 Hz and 40 Hz using a Hamming windowed sinc FIR filter. Then, artifacts were automatically removed using the Artifact Subspace Recostruction (ASR) algorithm, with a standard deviation cutoff for the removal of bursts *k* = 20, in order to achieve a conservative reconstruction while keeping in line with the most recent literature on pre-processing developmental EEG data [37]. Then, the data were segmented into epochs of 1500 ms, including a baseline of 200 ms (−200 to 1300 ms), time-locked to the stimulus onset and baseline-corrected. Finally, a motion artifact detection routine was carried out in order to detect residual artifacts. In particular, epochs were excluded automatically if they had an amplitude lower than −75 uV or greater than 75 uV, or a joint probability standard deviation larger than 3. Only standard trials were used for each condition. Furthermore, the first two trials of the sequence as well as trials directly following a deviant were excluded from the final analysis, to avoid strong dishabituation or novelty detection responses [9]. As a result of pre-processing and trial exclusion, the final dataset included on average across babies, 44 trials for the condition speech (range: 30–56) and 42 for the condition vocalization (range: 30–58). Grand-averaged ERPs are reported in Figure 4.

#### 2.4.3. NIRS-EEG Feature Extraction and Classification

**Figure 5:**
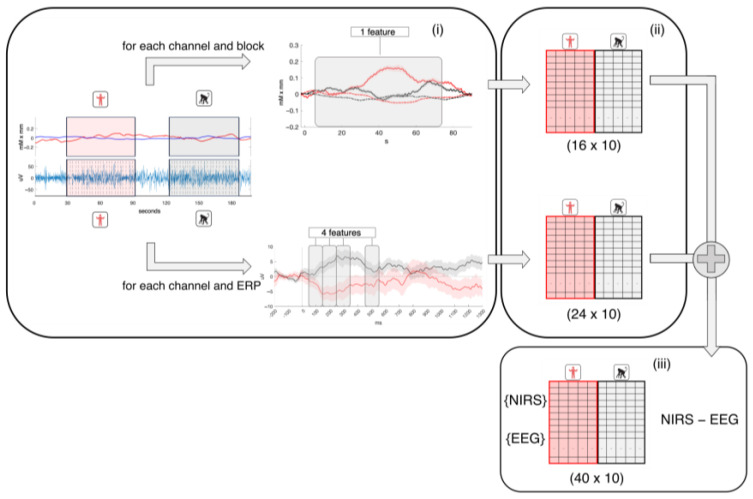
Schematic illustration of deriving the feature matrices. (**i**) Features were extracted from the EEG and NIRS signals separately, as described in Section 2.4.3. The left panel shows a portion of the NIRS signal on top (red: HbO, blue: HbR) and of the concurrent EEG at the bottom. The right panel shows an example of NIRS block and EEG trial across conditions (red: speech, black: vocalizations, with continuous lines representing HbO and dashed lines representing HbR, within the NIRS block). (**ii**) Each EEG trial in each condition thus contributed 4 features per channel, resulting in a vector of 26 features for each trial. Each NIRS block contributed 1 feature per channel, resulting in a vector of 16 features per each trial. (**iii**) NIRS and EEG submatrices were merged along the feature dimension, thus yielding an NIRS-EEG feature matrix of 40 features for each of the 5 trials in each condition, i.e., a total of 10 observations (56 features for the EEG+HbO+HbR classifier). After merging, the matrix was normalized and trials were classified. The final classification accuracy of each classifier was then statistically assessed against the chance distribution obtained by randomly relabeling trials (*n* = 100 times).

Figure 5 describes the workflow for feature extraction and classification. In particular, NIRS features were extracted for each block in each channel from both HbO and HbR, separately. Specifically, the hemodynamic response was averaged within a time window starting at the onset of the stimulus and ending 85 s after onset. This way, each channel contributed one feature for each block, yielding a total of 16 features × 10 blocks, i.e., 160 dimensions for HbO and HbR independently. An additional NIRS matrix was also created, with HbO and HbR features concatenated, with 32 features × 10 blocks.

EEG features were extracted for each epoch in each electrode in four time windows (50–150, 150–250, 250–350, 550–650 ms). These windows were chosen because they correspond to the P1-N1-P2-N2 auditory complex in infants [38,39]. Then, features from EEG epochs presented within the same NIRS block were averaged together. Thus, each epoch in each condition in each electrode contributed 4 features, yielding an EEG feature matrix of 24 × 10.

The multivariate pattern matrix was built by merging the NIRS and EEG matrices along the feature dimension, yielding a final matrix of 40 features (16 NIRS channels and 4 features for each of the 6 EEG channels) by 10 blocks. Additionally, an NIRS-EEG matrix was also computed concatenating EEG with both HbO and HbR features, with 56 × 10 dimensions.

After concatenation, feature vectors were normalized to have zero mean and unit variance [25]. Classification of multimodal trials, i.e., EEG averaged epochs/NIRS blocks, was performed using a toolbox by Ashton and colleagues [29], available online. Specifically, the original toolbox was designed to classify EEG epochs using time points or other features of interest. It employs a 4-fold cross-validation procedure in which 75% of the trials are used for training and the remaining 25% for testing [40]. The partitioning of trials is performed randomly a number of times (*n* = 200) and within each fold of cross-validation, trials are averaged to obtain a single *pseudotrial* per condition, per each fold. The rationale for this procedure is that it reduces the noise normally present in single trial data and improves classification accuracy. Finally, classification is carried out using Linear Support Vector Machine (SVM). Using synthetic fNIRS data, SVMs were shown to perform similarly to Linear Discriminant Analysis under most conditions, but they were significantly better at high levels of noise [41]. Recently, they were also shown to perform well in decoding EEG brain patterns elicited by different visual stimuli in 12–15-month-olds [42] and, in a recent systematic comparison, they were found to achieve better performance than more complex models [43], thus making them a standard choice when classifying neuroimaging data. For further details on the classification, we refer the reader to the original publication of the toolbox [29]. We obtained classification accuracies as the average over the number of iterations performed (*n* = 200). In the current study, we also used accuracies from each iteration to calculate the standard error of the average.

Classification was carried out on HbO NIRS data, HbR NIRS data, HbO+HbR, EEG data, and on combined HbO and EEG, HbR and EEG, and EEG and HbO+HbR. Monomodal data were used for baseline comparisons assessing whether and to what extent multimodal data provided better classification.

Statistical significance was assessed by comparing each classification accuracy to the distribution of accuracies obtained by randomly relabeling trials 100 times (“null ”accuracies”); *p* values were then obtained as the fraction of accuracies from the permutations that were equal to or greater than the true accuracy.

## 3. Results

**Figure 6:**
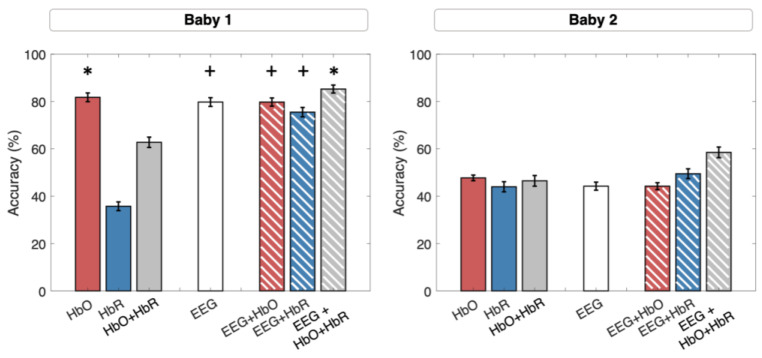

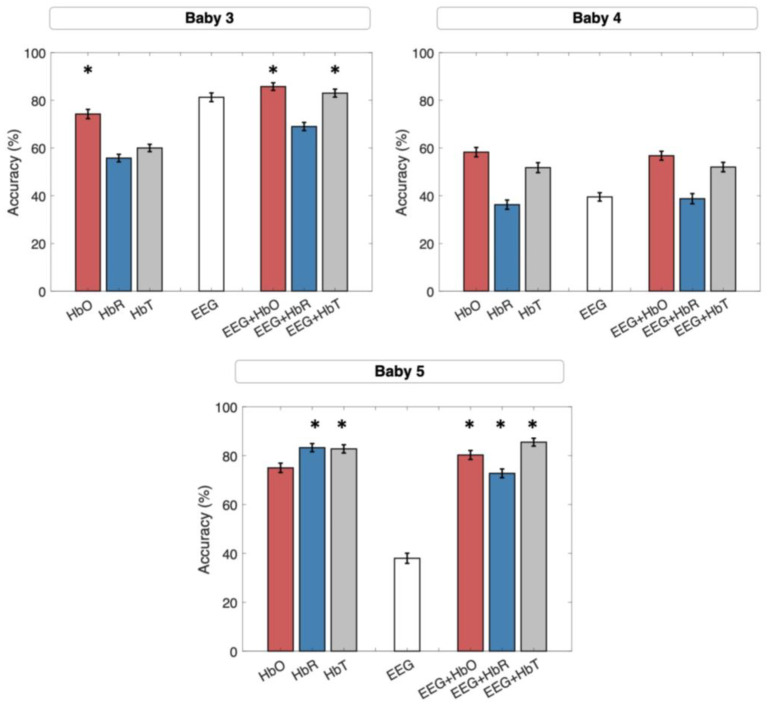
Classification accuracies achieved when using NIRS (HbO, HbR, HbO+HbR), EEG, and NIRS-EEG (HbO, HbR, HbO+HbR) for all babies. The height of the bar represents the average accuracy over classification iterations (*n* = 200), while the error bars represent its standard error. Asterisks mark statistical significance (*p* < 0.05), plus signs mark marginal significance (*p* < 0.1).

Figure 6 shows the obtained classification accuracies for each classification for all babies separately.

*NIRS data alone.* On average, across babies, when using only HbO the classifier reached a mean accuracy of 67.40% (SE: 6.2%); when using only HbR, it reached a mean accuracy of 51% (STD: 8.8%) and finally, when using both components, it reached a mean accuracy of 60.75% (STD: 6.2%). At subject-level, the NIRS-only classifiers performed significantly for Baby 1 (HbO: 81.7%, SE: 1.85%, chance level: 53.52%, *p* = 0.03), Baby 3 (HbO: 74.25%, SE: 1.94, chance level: 49.76, *p* = 0.04), and Baby 5 (HbO: 75%, SE = 1.9, *p* = 0.09; HbR: 83.25%, SE: 1.6, *p* = 0.04, HbO+HbR: 82.75%, SE = 1.6, *p* = 0.01).

*EEG data alone*. With EEG data, the classifier reached an average accuracy, across babies, of 56.55% (STD: 9.8%). At subject-level, the classifier performed significantly for Baby 1 (79.7%, SE: 1.8%, chance level: 48.47%, *p* = 0.06) and Baby 3 (81.25%, SE: 1.8, chance level: 50.7, *p* = 0.04).

*Combined NIRS and EEG data*. Combining the feature matrices from the two modalities achieved an average accuracy, across babies, of 69.35% (SE: 8.1), 61.10% (SE: 7.2), and 72.85% (SE: 7.2). At subject-level, the combined NIRS-EEG classifier performed significantly for Baby 1, in particular when using both hemoglobin components (EEG+HbO: 79.7%, SE: 1.7, chance level: 49.7%, *p* = 0.06; EEG+HbR: 75.5%, SE: 1.9, chance level: 48.2%, *p* = 0.07; EEG+HbO+HbR: 85.25%, SE: 1.7, *p* = 0.04), for Baby 3 (EEG+HbO: 85.75%, SE: 1.6, *p* = 0.02; EEG+HbR: 69%, SE: 1.7, p ns, EEG+HbO+HbR: 83%, SE = 1.68, *p* = 0.01), and for Baby 5 (EEG+HbO: 80.25%, SE = 1.8, *p* = 0.04; EEG+HbR: 72.75%, SE = 1.8, *p* = 0.06, EEG+HbO+HbR: 85.50%, SE = 1.6, *p* = 0.03).

**Figure 7:**
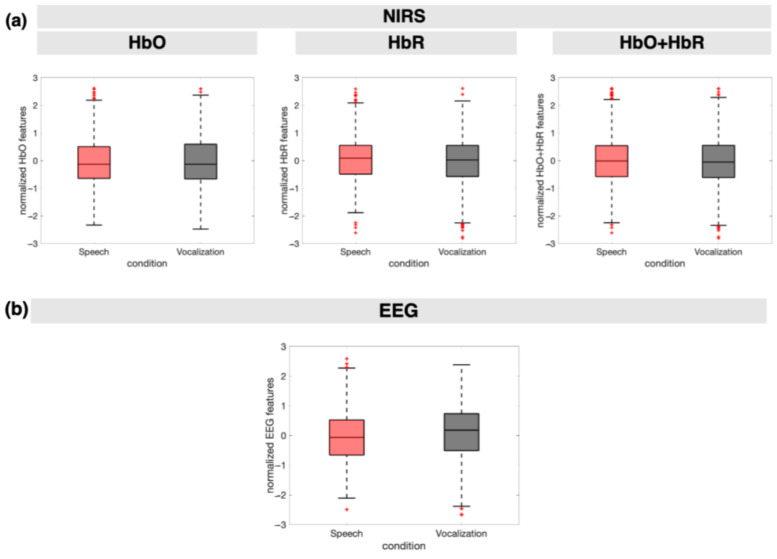

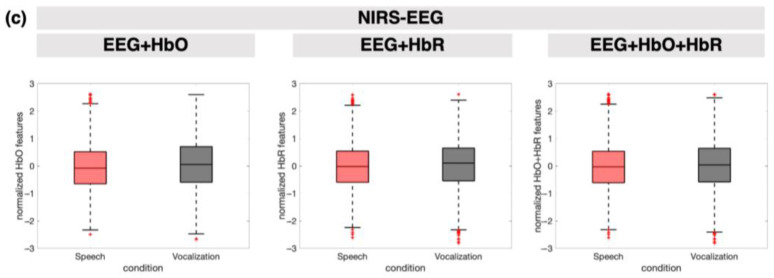
Discriminability of speech from monkey vocalization trials based on the chosen features: (**a**) Boxplots of NIRS features from all channels, for the two conditions, from all babies; (**b**) Boxplots of EEG features from all channels, for the two conditions, from all babies; (**c**) Boxplots of NIRS-EEG features from all channels, for the two conditions, from all babies. In all boxes, the central line represents the mean value of the distribution, and boxes extend from the 25th to the 75th percentiles, and the black whiskers extend to the most extreme data points not considered outliers (which are marked with red crosses). Features, extracted as the averages of blocks for NIRS and epochs’ time windows for EEG, as described in Section 2.4.3, are shown here on the *y*-axis after normalization.

Figure 7 shows the distributions of the employed NIRS and EEG features.

## 4. Discussion

In this work, we presented a multivariate pattern analysis (MVPA) pipeline applied to combined NIRS-EEG data, simultaneously recorded from two newborn infants listening to human speech and monkey vocalizations.

This is, to our knowledge, the first study investigating the feasibility of classifying NIRS-EEG features derived from infant data with an MVPA classifier at the single subject level. Within-subject classification of infant data is challenging in part, because inter-subject variability is often greater in infants than in adults [44] due to individual, but also maturational, differences [45].

Indeed, classification accuracies were quite different between the participants we tested. They were statistically significant for three out of five babies. For these babies, accuracies were already high when using either modality separately (Baby 1) or NIRS only (Babies 3 and 5). Nevertheless, for all of them, accuracies further increased when using EEG+HbO+HbR. In the case of non-successful decoding (Babies 2 and 4), no classification achieved above-chance accuracy, but the performance for Baby 2 was still the highest for EEG+HbO+HbR.

These diverging results are not surprising, as some participants may be more successful than others in discriminating between experimental conditions, even when using classical univariate statistical analyses. Classification accuracy captures these inter-individual differences. What is important from our perspective here is not the absolute level of accuracy achieved, but that combined EEG, HbO, and HbR, i.e., multimodal classification, yielded higher accuracies than monomodal classification.

It is interesting to notice that, for four out of five subjects, when both HbO and HbR are used in the full NIRS-EEG matrix, the resulting accuracies are higher than those achieved with any other dataset. This result is in line with previous findings from Gemignani and colleagues [46], showing how, on adult data, using both HbO and HbR in a discrimination routine yielded larger accuracies, and with smaller variation across the whole group, compared to using separate hemoglobin components, thus offering a more flexible analysis that better adapts to the individual’s own hemodynamic characteristics. 

This result suggests that using HbR in addition to HbO within a classification routine leverages stronger correlations between this hemoglobin component and the EEG signal. This conforms with previous work suggesting that this hemoglobin component displays a stronger correlation with EEG than HbO does [47,48], a point that warrants further investigation.

The technical challenge of the present work was to meaningfully concatenate NIRS and EEG matrices, each contributing a different number of trials. Given the different time scales of EEG and NIRS, the former naturally contributes more trials. Our work proposes to average features from the EEG epochs presented within an NIRS block so that the two modalities contribute the same number of trials. This approach is not without limitations: in particular, it results in a matrix with many more features than trials to classify. Future work taking this approach may thus add a feature selection step in order to reduce the number of features to be used, but also to optimize the complementarity between multimodal features, also by making use of ad-hoc synthetic datasets [49] Relatedly, future work should investigate the spatial specificity of the effect under investigation, in this case the perception of prosodic contours at birth, to learn whether some channels are more informative than others and so whether the classifier would benefit from restricting the set of channels to be included in the multivariate patterns. Finally, the applicability of the proposed method, and especially the advantage of using the fullest multimodal matrix, will need to be tested on a wider sample size of infants; a larger sample will also allow one to evaluate whether individual classification accuracies are somehow moderated by relevant methodological or by individual sources of variability.

## 5. Conclusions

This work presented a within-subject MVPA approach for the analysis of NIRS-EEG data: multimodal and multivariate patterns were concatenated and classified using linear SVMs. The proposed approach was tested on NIRS-EEG data acquired from five newborns while they listened to human speech and monkey vocalizations. Patterns elicited by the two types of stimuli were classified, using EEG, HbO, and HbR, in the same feature matrix. In three out of five infants, this combined multimodal classification achieved the highest accuracy. For the other infants, all classification routines resulted in non-significant classification accuracies, but for one of them the classifier combining EEG, HbO, and HbR still outperformed all separate-modality routines. This work demonstrates that leveraging both EEG and NIRS, especially both hemoglobin components, can meaningfully contribute to successfully classifying brain patterns elicited by different types of stimuli.

## Figures and Tables

**Table 1:** Mean acoustic measures (and standard deviations) of the Speech and Monkey Vocalization conditions.

	Speech	Vocalizations	*p* Value	95% CI
Duration (s)	0.64 (0.15)	0.64 (0.14)	0.55 (t = −0.607, df = 26)	[−0.003, 0.002]
Mean Pitch (Hz)	255.20 (41.23)	281.90 (101.91)	0.18 (t = 1.356, df = 26)	[−13.757, 67.086]

## Data Availability

Given the sensitive nature of the biomedical data involved, all data are available from the authors upon reasonable request.
